# Screening of Depression in Elderly Population Using a Geriatric Depression Scale in the Field Practice Area of Urban Health Training Centre Attached to SMS Medical College, Jaipur

**DOI:** 10.7759/cureus.15859

**Published:** 2021-06-23

**Authors:** Abhishek Kumar, Dilip Raj, Ajay Gupta, Amit Kumar

**Affiliations:** 1 Community Medicine, Sawai Man Singh (SMS) Medical College, Jaipur, IND; 2 Community Medicine, Indira Gandhi Institute of Medical Sciences, Patna, IND

**Keywords:** depression, elderly, prevalence, screening, urban, geriatric depression scale

## Abstract

Background and objective: Depression is one of the most common illnesses worldwide, with more than 264 million people affected. Various studies in the elderly population have estimated the prevalence of depression across India, with results ranging from 6% to 62%. The objectives of this study were to estimate the prevalence of depression among the elderly population using a Geriatric Depression Scale (GDS) and to find out the association between various sociodemographic parameters and depression among elderly people.

Materials and methods: This cross-sectional study was conducted in the field practice area of Urban Health Training Centre (UHTC), attached to Sawai Man Singh (SMS) Medical College, Jaipur from September 2019 to July 2020 among elderly people. Some 250 participants were interviewed.

Results: Depression was present in 41.6% of the study participants. Age group, gender, marital status, educational qualification, type of family, financial dependence, socioeconomic status, and presence of morbidity were significantly associated with the presence of depression among study participants (p<0.05).

Conclusion: The prevalence of depression in the urban elderly population was high (41.6%). By identifying risk factors for depression among the elderly population and screening them on time, we can reduce the severity and burden of the disease to a greater extent.

## Introduction

An increase in life expectancy is an outstanding achievement of this century all over the world, but this has brought with it new public health challenges. It means that elderly people are living longer and that demographic changes are being reflected in the age pyramids.

Aging being a normal multidimensional process is associated with physical, social, and psychological changes [1]. The four areas where the impact is being felt the most are health, economy, social and political, due to an aging population. This increase in the number of elderly persons has put an extra burden on the health care and social care systems in the country. Old age comes with a lot of different health concerns and diseases. The most common mental and neurological disorders in this age group are dementia and depression, which affect approximately 5% and 7% of the world’s older population, respectively [2]. Urbanization, nuclearization of family, migration, and career-oriented families are making care of the elderly people neglected to a large extent, making them more prone to depression. Depression is one of the most common illness worldwide, with more than 264 million people affected [3]. Currently, depression is the third leading contributor to the global disease burden and will rise to first place by 2030 [4] but stands second in terms of overall psychiatric disorders. Depression arises due to a complex interaction of social, psychological, and biological factors. Geriatric mood disorders lead to suffering, thereby increase the burden on the healthcare system, deteriorate the prognosis of many medical conditions, and contribute to disability. Added to this, depression also increases the false perception of poor health and the overutilization of medical services. Depression in the elderly is yet to receive its recognition in India and due to this only a few community-based studies have been conducted in India so far to address this issue [5]. Various studies in the elderly population have estimated the prevalence of depression across India, with results ranging from 6% to 62%. Methodological differences may be the reason for such a huge variation in prevalence [6-7]. Keeping this in mind, the different above-mentioned problems of the elderly, the need was strongly felt to assess the prevalence of depression and its determinants among elderly people, to plan regionally sensitive intervention strategies for engaging, and empowering the elderly population against depression.

The objectives of this study were to estimate the prevalence of depression among the elderly population using a Geriatric Depression Scale (GDS) and to find out the association between various sociodemographic parameters and depression among elderly people.

## Materials and methods

This cross-sectional study was conducted in the field practice area of Urban Health Training Centre (UHTC), attached to Sawai Man Singh (SMS) Medical College, Jaipur from September 2019 to July 2020 among elderly people.

Individuals aged 60 years and above of either of the sex residing in that area for a period of at least one year prior to start of the study and scoring more than or equal to 20 on mini-mental state examination Hindi version (MMSE Hindi version) were included in the study. Persons having severe hearing loss, aphasia, mental retardation, and a score of less than 20 on the mini-mental state examination were excluded. Persons from locked houses and could not be contacted even after two visits were also excluded from the study.

Considering the prevalence of depression among elderly people in the urban population as 15.6% [8], with an absolute error of 5%, confidence level of 95%, and taking 10 % nonresponse rate, the sample size was estimated to be 229. Finally, 250 participants were interviewed.

The necessary approval was obtained from the Institutional Ethics Committee (IEC) of SMS Medical college prior to commencement of the study. Under the field practicing area of UHTC, Sushilpura was selected for study purposes, which has a population of 6000. According to SRS Statistical Report 2016, the proportion of older adults in the Indian population is about 8.3% [9]. Considering this, the expected elderly population in the study area was around 498, which was sufficient for obtaining the required sample size for the study. For sample size collection, initially, one house was selected randomly. As the distribution of the elderly population in the mentioned area is not well defined, therefore every alternate house, from the first selected house, was visited till the required sample size was achieved. If one house was having more than one elder person, then all of them were included. After explaining the nature of this study, informal verbal consent was obtained from each one of the participants and the face-face interview was conducted using a pre-designed, semi-structured schedule which contains:

- Information on socio-demographic profile of the respondent like age, sex, educational status, type of family, family income, and the number of family members residing in the same house.

- Mini-mental state examination (MMSE) Hindi version [10] was applied to check the cognition of each participant and it is used as inclusion and exclusion criteria.

- GDS, first created by Yesavage et al., which is a 30 questions rating scale, was used to screen for depression: where a score of 0-9 was considered normal, score 10-19 implied mild depression, and score 20-30 indicated severe depression [11].

Data were entered into a Microsoft Excel spreadsheet and analyzed using SPSS 21 [trial version] (IBM Corp., Armonk, NY). Descriptive statistics were used and Pearson’s Chi-square test as the test of significance; taking a p-value of <0.05 as statistically significant.

## Results

**Figure 1:**
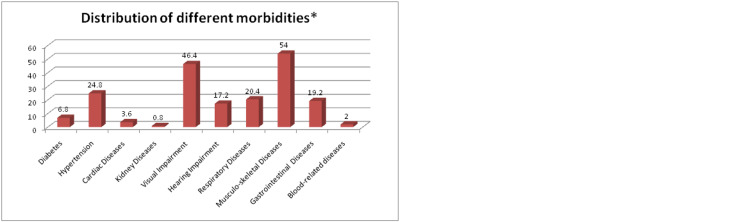
Distribution of different morbidities among study participants. *Multiple choice

In total 250 persons were interviewed. The maximum proportion of the study participants were in the age group of 60-69 years (59.2%) and the mean age of the participants was 67.9 years with a standard deviation of 7.08 years. The majority of the participants were male (53.2%), married (69.6%), and belonged to the Hindu community (96.8%). Around 68% were illiterate, belonged to the joint family (79.2%), were financially dependent (57.2%), and belonged to the upper lower class (34.8%) according to Modified Kuppuswamy socioeconomic scale 2021 [12]. Morbidities were present in 76.8% of the study participants (Table 1), with musculoskeletal diseases (54%) being the most common morbidity, followed by visual impairment (46.4%), hypertension (24.8%), and respiratory illness (20.4%) (Figure 1).

**Table 1:** Sociodemographic profiles of study participants (N=250).

Characteristics	Frequency (n)	Percentage
Age group		
60-69	148	59.2
70-79	79	31.6
80-89	21	8.4
90-99	2	0.8
Gender		
Male	133	53.2
Female	117	46.8
Marital status		
Married	174	69.6
Divorced	2	0.8
Widow	74	29.6
Education		
Illiterate	172	68.8
Till 8th	49	19.6
Till 10th	20	8
Till 12th	5	2
Graduate	4	1.6
Type of family		
Nuclear	52	20.8
Joint	198	79.2
Financial dependency		
Dependent	143	57.2
Independent	107	42.8
Socioeconomic status		
Upper	15	6
Upper middle	30	12
Lower middle	47	18.8
Upper lower	87	34.8
Lower	71	28.4
Morbidity		
Present	192	76.8
Absent	58	23.2
Religion		
Hindu	242	96.8
Muslim	8	3.2

 

 

**Figure 2:**
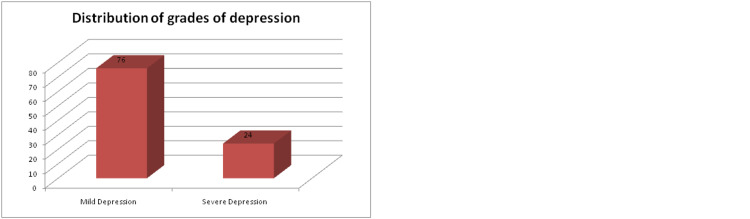
Distribution of grades of depression among study participants (N=104).

Depression was present in 41.6% of the study participants (Table 2). Mild depression was most common (76%), followed by severe depression (24%). The mean GDS score was 9.5 with a standard deviation of 6.4 (Figure 2).

**Table 2:** Prevalence of depression among the study participants (N=250).

Depression	Frequency	Percentage
Present	104	41.6
Absent	146	58.4
Total	250	100

The proportion of elderly with depression was higher in the age group of 80 years and above (69.6%), female gender (54.7%), divorced (100%), illiterates (50%), nuclear family (55.8%), financially dependence (48.3%), lower socioeconomic class (49.3%) and with morbidities (51%). Age group, gender, marital status, educational qualification, type of family, financial dependence, socioeconomic status, and presence of morbidity were significantly associated with the presence of depression among study participants (p<0.05) (Table 3).

**Table 3:** Association of depression with different characteristics of study population (N=250).

Characteristics		Depression		No depression		p-value
		N	%	N	%	
Age group	60-69	48	32.4	100	67.6	<0.05
	70-79	40	50.6	39	49.4	
	80 and above	16	69.6	7	30.4	
Gender	Male	40	30.1	93	69.9	<0.05
	Female	64	54.7	53	45.3	
Marital status	Married	56	32.2	118	67.8	<0.05
	Divorced	2	100	0	0	
	Widowed	46	62.2	28	37.8	
Religion	Hindu	100	41.3	142	58.7	>0.05
	Muslim	4	50	4	50	
Educational qualification	Illiterate	86	50	86	50	<0.05
	Upto primary	13	26.5	36	73.5	
	Upto 10th	4	20	16	80	
	Upto 12th	1	20	4	80	
	Graduate	0	0	4	100	
Type of family	Nuclear	29	55.8	23	44.2	<0.05
	Joint	75	37.9	123	62.1	
Financial dependency	Dependent	69	48.3	74	51.7	<0.05
	Independent	35	32.7	72	67.3	
Socioeconomic status	Upper class	3	20	12	80	<0.05
	Upper middle class	5	16.7	25	83.3	
	Lower middle class	19	40.4	28	59.6	
	Upper lower class	42	48.3	45	51.7	
	Lower class	35	49.3	36	50.7	
Morbidity status	Present	98	51	94	49	<0.05
	Absent	6	10.3	52	89.7	

## Discussion

Prompt recognition and early treatment of mental, neurological, and substance use disorders in older adults are essential. Both psychosocial interventions and medicines are recommended simultaneously. The present study was conducted to know the magnitude of depression and its associated factors among 250 geriatric study subjects residing in the UHTC field practice area of Jaipur city.

In the present study, the mean age of the participants was 67.9 ± 7.08 years, which was consistent with the findings of a study conducted by Chawla et al. [13]. Maximum proportions of the respondents were in the age group of 60-69 years (59.2%) in the present study. Similar findings were noted from others studies like Nair et al. [14] and Nautiyal et al. [15].

In the present study, the majority of the study participants (76.8%) were having one or more morbidities. This finding was slightly higher than the findings reported by Gupta et al. [10] and Mullick et al. [16] which showed 59% and 62% participants with morbidity respectively. It was noted in our study that musculoskeletal diseases (54%) were the most common morbidity present in the geriatric population, followed by visual impairment (46.4%), which was different from the findings of other studies like, Mullick et al. [16], who found cardiovascular system (CVS) (31.7%) as the most common related morbidity, followed by cataract (27.47%). This can be attributed to the fact that the majority of the participants in our study belonged to the lower socioeconomic strata who were primarily daily wage laborers.

The prevalence of depression in the present study was 41.6%. This may be affected due to ongoing coronavirus disease 2019 (Covid-19) pandemic and country-wide lockdown during the study period. Similar results were found in the study conducted by Kavithai et al. in rural areas of Puducherry [17] which found the prevalence of depression to be 41.4%. Several other studies reported a lower prevalence of depression as compared to our study like Chawla et al., Nair et al., Nautiyal et al., and Konda et al. as 22.72%, 32.4%, 29.94%, and 23%, respectively [13-15, 18]. This observed difference in the finding could be attributed to different sampling techniques, sample sizes, study settings, and instruments used.

In the present study, it was found that there was a significant association of depression with the increasing age of geriatric persons. The age group of 80 years and above was found to be more under depression (69.6%) than other age groups. Advancing age is often accompanied by various events in life such as the death of a spouse, retirement, financial dependence, etc. Similar findings were observed by certain other studies [1, 10, 14]. However, Kavithai et al. reported in their study that depression was more common among those aged below 80 years [17]. The current study showed that gender was significantly associated with the prevalence of depression. Observation showed that females (54.7%) were more depressed than the male (30.1%) population, which was very similar to the findings of Mullick et al. [16], but few results were contrary to this finding like Nautiyal et al. [15], and Mandolikar et al. [19], which found males were more depressed than females.

Marital status had a significant association with the prevalence of depression in our study. Divorced and widowed had a higher prevalence of depression compared to those who were married. This was in concordance with the findings of studies conducted by Gupta et al. [10] and Naveen et al. [20].

We found that there was a significant association between educational qualification and prevalence of depression among the respondents with illiterate persons being more depressed. Similar results were noted from different studies like Rathod et al. [1], Sanjay et al. [21], and Paul et al. [22].

The present study also depicted that the prevalence of depression is significantly associated with the type of family, having nuclear type more associated with depression and few studies had found similar results like Chawla et al. [13] and Konda et al. [18]. This may be explained by the fact that in nuclear families, sometimes the level of support and care of elderly people may be low or even missing, which ultimately may give rise to depression. Contrary to this, Sanjay et al. [21] found that people belonging to the joint type of family were more depressed.

This study showed that financial dependency was significantly associated with the prevalence of depression. Similar findings were noted from studies like Mullick et al. [16] and Pilania et al. [23]. With the loss of a job or retirement, older people often feel the loss of dignity and self-respect. This can result in feelings of loneliness or psychological distress. Socioeconomic status had a significant association with the prevalence of depression in our study and it was the highest in the lower class. Similar findings have been noted from studies conducted in other settings [14, 21-22]. People belonging to lower socioeconomic strata are often striving hard to meet their daily needs. They have limited access to many social activities because of financial constraints. So, they end up getting depression as a result.

In the present study, there was a significant association observed between morbidity and prevalence of depression, having the presence of morbidities more commonly associated with depression. Several other studies supported the fact [13, 18, 23]. These comorbidities along with depression increase physical disability, poor compliance, and increased healthcare utilization leading to poor quality of life and further complicating the treatment of depression [21].

Our study had certain limitations. The main limitation of the study is that being cross-sectional in nature, it does not permit determination of the temporality of the relationship between the various factors and depression. Due to the ongoing COVID-19 pandemic, few were reluctant in giving their response and tried their best to get rid of the interview as early as possible, and that might have affected their response by adding bias. GDSAQ is a screening tool for depression; hence confirmed diagnosis could not be established.

## Conclusions

The prevalence of depression in the urban elderly population was high (41.6%). Depression increased with age and was associated with female gender, illiteracy, lower socioeconomic status, financial dependence, and presence of comorbidities. By identifying risk factors for depression among the elderly population and screening them on time, we can reduce the severity of the disease to a greater extent, which in turn will reduce the burden on the healthcare system, as well as the catastrophic health expenditure of the family. It can be best done by educating the family members and providing special training to the medical officers, auxiliary nurse midwife (ANM), and accredited social health activist (ASHA) for diagnosing psychiatric illness at the community level.
